# Diaqua­bis­(2-oxo-2*H*-chromene-3-carboxyl­ato)zinc(II)

**DOI:** 10.1107/S1600536810050865

**Published:** 2010-12-11

**Authors:** Yue Cui, Qian Gao, Huan-Huan Wang, Lin Wang, Ya-Bo Xie

**Affiliations:** a College of Environmental and Energy Engineering, Beijing University of Technology, Beijing 100124, People’s Republic of China

## Abstract

In the title compound, [Zn(C_10_H_5_O_4_)_2_(H_2_O)_2_], the Zn^II^ atom lies on a crystallographic inversion center and is six-coordinated by two O atoms from water mol­ecules in the axial positions and four O atoms from two deprotonated coumarin-3-carb­oxy­late ligands in the equatorial plane, forming a slightly distorted octa­hedral coordination geometry. O—H⋯O hydrogen-bonding inter­actions involving the water mol­ecules form infinite chains parallel to [010].

## Related literature

For related structures, see: Chu *et al.* (2010 ▶). For hydrogen-bond motifs, see: Bernstein *et al.* (1995 ▶); Etter (1990 ▶).
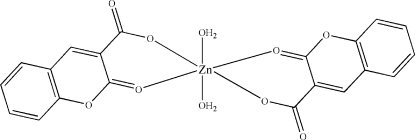


## Experimental

### 

#### Crystal data

[Zn(C_10_H_5_O_4_)_2_(H_2_O)_2_]
*M* *_r_* = 479.70
Triclinic, 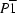

*a* = 6.6113 (13) Å
*b* = 6.8404 (14) Å
*c* = 10.392 (2) Å
α = 85.64 (3)°
β = 89.47 (3)°
γ = 66.09 (3)°
*V* = 428.27 (18) Å^3^
*Z* = 1
Mo *K*α radiation
μ = 1.50 mm^−1^
*T* = 293 K
0.2 × 0.2 × 0.2 mm

#### Data collection

Bruker APEXII CCD diffractometer
Absorption correction: multi-scan (*SADABS*; Bruker, 2005 ▶) *T*_min_ = 0.741, *T*_max_ = 0.748
2642 measured reflections
1808 independent reflections
1793 reflections with *I* > 2σ(*I*)
*R*_int_ = 0.013

#### Refinement

*R*[*F* ^2^ > 2σ(*F* ^2^)] = 0.022
*wR*(*F* ^2^) = 0.061
*S* = 1.12
1808 reflections
142 parameters
H-atom parameters constrained
Δρ_max_ = 0.42 e Å^−3^
Δρ_min_ = −0.28 e Å^−3^

### 

Data collection: *APEX2* (Bruker, 2005 ▶); cell refinement: *SAINT* (Bruker, 2005 ▶); data reduction: *SAINT*; program(s) used to solve structure: *SHELXS97* (Sheldrick, 2008 ▶); program(s) used to refine structure: *SHELXL97* (Sheldrick, 2008 ▶); molecular graphics: *ORTEPIII* (Burnett & Johnson, 1996 ▶), *ORTEP-3 for Windows* (Farrugia, 1997 ▶) and *PLATON* (Spek, 2009 ▶); software used to prepare material for publication: *SHELXTL* (Sheldrick, 2008 ▶).

## Supplementary Material

Crystal structure: contains datablocks global, I. DOI: 10.1107/S1600536810050865/dn2633sup1.cif

Structure factors: contains datablocks I. DOI: 10.1107/S1600536810050865/dn2633Isup2.hkl

Additional supplementary materials:  crystallographic information; 3D view; checkCIF report

## Figures and Tables

**Table 1:** Hydrogen-bond geometry (Å, °)

*D*—H⋯*A*	*D*—H	H⋯*A*	*D*⋯*A*	*D*—H⋯*A*
O1*W*—H1⋯O3^i^	0.82	1.88	2.6950 (17)	179
O1*W*—H2⋯O3^ii^	0.93	1.83	2.7473 (19)	168

Symmetry codes: (i) 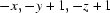; (ii) 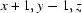.

**Fig. 1.**
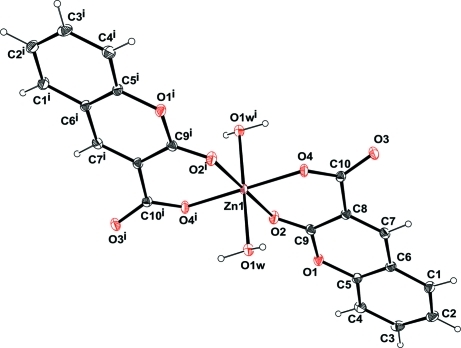
Molecular view of (I) with the atom-labelling scheme. Displacement ellipsoids are drawn at the 50% probability level. H atoms are represented as small spheres of arbitrary radii. [Symmetry code: (i) -x+1, -y+1, -z+1]

**Fig. 2.**
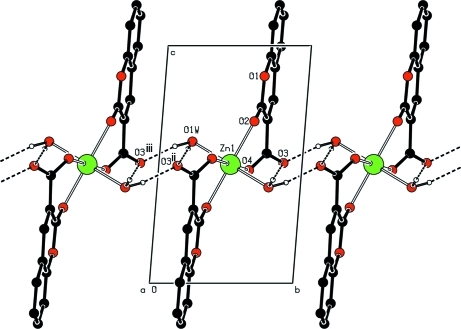
Partial packing view of compound ( I ), showing the formation of chains along [010] built from hydrogen bonds, and the formation of R 24(8) rings. For the sake of clarity, H atoms not involved in hydrogen bonding have been omitted. [Symmetry codes: (ii) -x, -y+1, -z+1; (iii) x+1, y-1, z]

## supplementary crystallographic information

### Comment

In the past decades, numerous papers dealing with mononuclear zinc complexes have been published (Chu *et al.* 2010). Herein, we report the synthesis and crystal structure of a new mononuclear zinc complex.

In the title compound, [Zn(C_10_H_5_O_4_)_2_(H_2_O)_2_], each Zn^II^ atom lies on a crystallographic inversion center and is six-coordinated by two O atoms from water molecules in the axial positions and four O atoms from two deprotonated coumarin-3-carboxylic acid ligands in the equatorial plane, forming an octahedral coordination geometry (Fig. 1). O—H···O hydrogen bonds involving the water molecules build up chain parllel to the [0 1 0] axis (Table 1, Fig. 2). The O-H···O interactions results in the formation of R^4^_2_(8) rings (Etter, 1990; Bernstein *et al.*, 1995).

### Experimental

The title complex was synthesized by carefully layering a solution of ZnSO_4_.7H_2_O (28.8 mg, 0.1 mmol) in ethanol solution (10 ml) on top of a solution of coumarin-3-carboxylic acid (19.0 mg, 0.1 mmol) and LiOH (8.4 mg, 0.2 mmol) in H_2_O (10 ml) in a test-tube. After about one month at room temperature, colorless block-shaped single crystals suitable for X-ray investigation appeared at the boundary between ethanol solution and water with a yield of 27%.

### Refinement

The H atoms were placed geometrically (C—H = 0.93 Å) and treated as riding with *U*_iso(H)_ = 1.2_eq_(C) . H atoms of water molecule were located in difference Fourier maps and included in the subsequent refinement using restraints (O-H= 0.85 (1)Å and H···H= 1.40 (2)Å) with U_iso_(H) = 1.5U_eq_(O). In the last cycle of refinement they were treated as riding on their parent O atom.

### Crystal data

**Table:**

[Zn(C_10_H_5_O_4_)_2_(H_2_O)_2_]	*Z* = 1
*M**_r_* = 479.70	*F*(000) = 244
Triclinic, *P*1	*D*_x_ = 1.860 Mg m^−^^3^
Hall symbol: -P 1	Mo *K*α radiation, λ = 0.71073 Å
*a* = 6.6113 (13) Å	Cell parameters from 2271 reflections
*b* = 6.8404 (14) Å	θ = 3.4–28.4°
*c* = 10.392 (2) Å	µ = 1.50 mm^−^^1^
α = 85.64 (3)°	*T* = 293 K
β = 89.47 (3)°	Block, colourless
γ = 66.09 (3)°	0.2 × 0.2 × 0.2 mm
*V* = 428.27 (18) Å^3^	

### Data collection

**Table:**

Bruker APEXII CCD diffractometer	1808 independent reflections
Radiation source: fine-focus sealed tube	1793 reflections with *I* > 2σ(*I*)
graphite	*R*_int_ = 0.013
φ and ω scans	θ_max_ = 27.1°, θ_min_ = 3.4°
Absorption correction: multi-scan (*SADABS*; Sheldrick, 2008)	*h* = −8→8
*T*_min_ = 0.741, *T*_max_ = 0.748	*k* = −8→8
2642 measured reflections	*l* = 0→13

### Refinement

**Table:**

Refinement on *F*^2^	Primary atom site location: structure-invariant direct methods
Least-squares matrix: full	Secondary atom site location: difference Fourier map
*R*[*F*^2^ > 2σ(*F*^2^)] = 0.022	Hydrogen site location: inferred from neighbouring sites
*wR*(*F*^2^) = 0.061	H-atom parameters constrained
*S* = 1.12	*w* = 1/[σ^2^(*F*_o_^2^) + (0.0216*P*)^2^ + 0.2757*P*] where *P* = (*F*_o_^2^ + 2*F*_c_^2^)/3
1808 reflections	(Δ/σ)_max_ < 0.001
142 parameters	Δρ_max_ = 0.42 e Å^−^^3^
0 restraints	Δρ_min_ = −0.28 e Å^−^^3^

### Special details

**Table:**

Geometry. All esds (except the esd in the dihedral angle between two l.s. planes) are estimated using the full covariance matrix. The cell esds are taken into account individually in the estimation of esds in distances, angles and torsion angles; correlations between esds in cell parameters are only used when they are defined by crystal symmetry. An approximate (isotropic) treatment of cell esds is used for estimating esds involving l.s. planes.
Refinement. Refinement of *F*^2^ against ALL reflections. The weighted *R*-factor *wR* and goodness of fit *S* are based on *F*^2^, conventional *R*-factors *R* are based on *F*, with *F* set to zero for negative *F*^2^. The threshold expression of *F*^2^ > σ(*F*^2^) is used only for calculating *R*-factors(gt) *etc*. and is not relevant to the choice of reflections for refinement. *R*-factors based on *F*^2^ are statistically about twice as large as those based on *F*, and *R*- factors based on ALL data will be even larger.

### Fractional atomic coordinates and isotropic or equivalent isotropic displacement parameters (Å^2^)

**Table:**

	*x*	*y*	*z*	*U*_iso_*/*U*_eq_	
Zn1	0.5000	0.5000	0.5000	0.01047 (10)	
O1	0.30343 (17)	0.68964 (17)	0.87151 (10)	0.0127 (2)	
O1W	0.49536 (17)	0.22209 (17)	0.59641 (10)	0.0133 (2)	
H1	0.3972	0.1951	0.5649	0.020*	
H2	0.6183	0.1022	0.5763	0.020*	
O2	0.45061 (18)	0.64366 (18)	0.68157 (10)	0.0141 (2)	
O3	−0.17320 (17)	0.86870 (17)	0.50573 (10)	0.0134 (2)	
O4	0.16868 (17)	0.63230 (18)	0.47583 (10)	0.0132 (2)	
C1	−0.2479 (3)	0.7880 (2)	0.99672 (15)	0.0146 (3)	
H1A	−0.3880	0.8073	0.9679	0.018*	
C2	−0.2071 (3)	0.7889 (3)	1.12660 (15)	0.0168 (3)	
H3A	−0.3203	0.8111	1.1850	0.020*	
C3	0.0042 (3)	0.7564 (2)	1.17064 (15)	0.0161 (3)	
H2A	0.0305	0.7567	1.2584	0.019*	
C4	0.1753 (3)	0.7237 (2)	1.08507 (15)	0.0148 (3)	
H11A	0.3160	0.7015	1.1143	0.018*	
C5	0.1306 (2)	0.7250 (2)	0.95493 (14)	0.0119 (3)	
C6	−0.0780 (2)	0.7579 (2)	0.90792 (14)	0.0122 (3)	
C7	−0.1088 (2)	0.7641 (2)	0.77180 (14)	0.0120 (3)	
H7A	−0.2480	0.7893	0.7385	0.014*	
C8	0.0600 (2)	0.7342 (2)	0.68973 (14)	0.0109 (3)	
C9	0.2798 (2)	0.6861 (2)	0.74172 (14)	0.0113 (3)	
C10	0.0171 (2)	0.7468 (2)	0.54635 (14)	0.0105 (3)	

### Atomic displacement parameters (Å^2^)

**Table:**

	*U*^11^	*U*^22^	*U*^33^	*U*^12^	*U*^13^	*U*^23^
Zn1	0.00736 (13)	0.01189 (14)	0.01013 (13)	−0.00175 (9)	0.00122 (8)	−0.00152 (9)
O1	0.0106 (5)	0.0164 (5)	0.0101 (5)	−0.0041 (4)	0.0009 (4)	−0.0028 (4)
O1W	0.0105 (5)	0.0139 (5)	0.0143 (5)	−0.0036 (4)	0.0005 (4)	−0.0016 (4)
O2	0.0099 (5)	0.0185 (5)	0.0133 (5)	−0.0048 (4)	0.0021 (4)	−0.0041 (4)
O3	0.0093 (5)	0.0143 (5)	0.0138 (5)	−0.0018 (4)	−0.0009 (4)	−0.0012 (4)
O4	0.0091 (5)	0.0168 (5)	0.0116 (5)	−0.0026 (4)	0.0013 (4)	−0.0032 (4)
C1	0.0139 (7)	0.0135 (7)	0.0155 (7)	−0.0046 (6)	0.0028 (6)	−0.0014 (6)
C2	0.0204 (8)	0.0135 (7)	0.0147 (7)	−0.0051 (6)	0.0069 (6)	−0.0015 (6)
C3	0.0247 (8)	0.0122 (7)	0.0098 (7)	−0.0059 (6)	0.0016 (6)	−0.0012 (5)
C4	0.0166 (7)	0.0133 (7)	0.0129 (7)	−0.0043 (6)	−0.0009 (6)	−0.0014 (6)
C5	0.0129 (7)	0.0092 (6)	0.0117 (7)	−0.0024 (5)	0.0034 (5)	−0.0017 (5)
C6	0.0133 (7)	0.0095 (6)	0.0129 (7)	−0.0036 (5)	0.0020 (5)	−0.0008 (5)
C7	0.0110 (7)	0.0108 (7)	0.0135 (7)	−0.0037 (5)	−0.0002 (5)	−0.0008 (5)
C8	0.0105 (7)	0.0100 (6)	0.0113 (6)	−0.0032 (5)	0.0004 (5)	−0.0016 (5)
C9	0.0123 (7)	0.0096 (6)	0.0105 (6)	−0.0026 (5)	0.0004 (5)	−0.0015 (5)
C10	0.0097 (6)	0.0108 (6)	0.0120 (7)	−0.0051 (5)	0.0006 (5)	−0.0009 (5)

### Geometric parameters (Å, °)

**Table:**

Zn1—O4	2.0122 (12)	C1—C6	1.406 (2)
Zn1—O4^i^	2.0122 (12)	C1—H1A	0.9300
Zn1—O1W^i^	2.0918 (12)	C2—C3	1.398 (2)
Zn1—O1W	2.0918 (12)	C2—H3A	0.9300
Zn1—O2	2.1548 (12)	C3—C4	1.388 (2)
Zn1—O2^i^	2.1548 (12)	C3—H2A	0.9300
O1—C9	1.3623 (18)	C4—C5	1.386 (2)
O1—C5	1.3801 (18)	C4—H11A	0.9300
O1W—H1	0.8200	C5—C6	1.391 (2)
O1W—H2	0.9269	C6—C7	1.426 (2)
O2—C9	1.2241 (18)	C7—C8	1.356 (2)
O3—C10	1.2501 (19)	C7—H7A	0.9300
O4—C10	1.2617 (19)	C8—C9	1.454 (2)
C1—C2	1.380 (2)	C8—C10	1.509 (2)
			
O4—Zn1—O4^i^	180.0	C3—C2—H3A	119.9
O4—Zn1—O1W^i^	88.05 (5)	C4—C3—C2	120.84 (15)
O4^i^—Zn1—O1W^i^	91.95 (5)	C4—C3—H2A	119.6
O4—Zn1—O1W	91.95 (5)	C2—C3—H2A	119.6
O4^i^—Zn1—O1W	88.05 (5)	C5—C4—C3	118.17 (15)
O1W^i^—Zn1—O1W	180.00 (3)	C5—C4—H11A	120.9
O4—Zn1—O2	87.20 (5)	C3—C4—H11A	120.9
O4^i^—Zn1—O2	92.80 (5)	O1—C5—C4	117.34 (14)
O1W^i^—Zn1—O2	90.89 (5)	O1—C5—C6	120.25 (13)
O1W—Zn1—O2	89.11 (5)	C4—C5—C6	122.41 (14)
O4—Zn1—O2^i^	92.80 (5)	C5—C6—C1	118.34 (14)
O4^i^—Zn1—O2^i^	87.20 (5)	C5—C6—C7	118.03 (14)
O1W^i^—Zn1—O2^i^	89.11 (5)	C1—C6—C7	123.62 (14)
O1W—Zn1—O2^i^	90.89 (5)	C8—C7—C6	121.59 (14)
O2—Zn1—O2^i^	180.000 (1)	C8—C7—H7A	119.2
C9—O1—C5	122.65 (12)	C6—C7—H7A	119.2
Zn1—O1W—H1	109.5	C7—C8—C9	119.36 (14)
Zn1—O1W—H2	110.7	C7—C8—C10	119.39 (13)
H1—O1W—H3	99.9	C9—C8—C10	121.24 (13)
C9—O2—Zn1	122.37 (10)	O2—C9—O1	114.78 (13)
C10—O4—Zn1	131.42 (10)	O2—C9—C8	127.30 (14)
C2—C1—C6	120.10 (15)	O1—C9—C8	117.92 (13)
C2—C1—H1A	119.9	O3—C10—O4	124.05 (14)
C6—C1—H1A	119.9	O3—C10—C8	116.41 (13)
C1—C2—C3	120.13 (15)	O4—C10—C8	119.51 (13)
C1—C2—H3A	119.9		

Symmetry codes: (i) −*x*+1, −*y*+1, −*z*+1.

### Hydrogen-bond geometry (Å, °)

**Table:**

*D*—H···*A*	*D*—H	H···*A*	*D*···*A*	*D*—H···*A*
O1W—H1···O3^ii^	0.82	1.88	2.6950 (17)	179.
O1W—H2···O3^iii^	0.93	1.83	2.7473 (19)	168.

Symmetry codes: (ii) −*x*, −*y*+1, −*z*+1; (iii) *x*+1, *y*−1, *z*.
